# The Limits of Medical Competition

**Published:** 1896-01-25

**Authors:** 


### The Hospital

An Institutional Journal of

The Medical Sciences and Hospital Administration, with

AN EXTRA NURSING SUPPLEMENT.

Vol. XIX.—No. 486.] [Jan. 18, 1896.

### The Limits of Medical Competition.

The decision of the General Medical Council, all too tardily arrived at, to "instruct its Penal Committee to inquire into cases of persons offending under Section 40 of the Medical Act (1858), and, where it sees fit, to instruct the legal advisers to proceed against such persons," draws attention to the whole question of medical competition, as it bears practically upon the interests of the public and of the medical profession. There can be no doubt that a very large section of the public has a great deal of sympathy with what are called "quacks."

The word "competition" has a kind of magical effect upon the minds of those trained in the school of modern political economy, and those who have sympathised with "quacks" have very generally done so under the impression that they were encouraging a healthy competition among different species of the same genus. Now, we have not one word to say against competition. It is a law of nature; an absolutely necessary condition of evolutionary progress. But we have two propositions to make which it seems to us may help to clear away a good deal of the light-excluding *débris* that has accumulated about the subject. The first is that there is not and cannot be any true and proper "competition" between the legally qualified medical man and the "quack"; and the second, that all needful and serviceable competition in the business of medical practice is provided for by the almost infinitely varied conditions of medical training, medical qualifications, and the prevailing methods of conducting their business adopted by different orders of medical men. Let us, in this connection, consider intelligently how the word "competition" is to be employed. There is a competition of life and a competition of work. As a graminivorous quadruped the sheep may be said to be a competitor of the horse; but in work the sheep is in no sense a competitor with the nobler animal. Nobody thinks of yoking a sheep to a cart or riding it to hounds, even though it has four legs and eats grass exactly like the horse. Similarly the quack may compete with the legally qualified man in his hunger for fees, and even in his skill in securing them. But in proper medical work, in the sciences of medicine, surgery, and physiology, in the art of diagnosing disease and scientifically treating it, he cannot really "compete" with the educated and legally examined and qualified doctor any more than the sheep can compete with the horse in the farmer's waggon or the hunting field. This is obvious; and no intelligent person who thinks for a moment will dream of questioning it.

Do medical men advance the foolish claim that they are to be protected against all competition? They do not. As a matter of fact there is no single calling in which competition is keener than it is in the medical profession; and there is no calling in which the benefits of competition are more certainly secured for the public. Why does the public always and everywhere encourage competition? For the one and only reason that it thereby secures cheaper and better goods, cheaper and better services, and a larger and more intrinsically valuable return for the money it lays out. Is it the case that the competition among legally qualified medical men is so universal and so keen as to compel them all to render to the public the highest possible services of which they are capable at the smallest possible cost? If it be, then we maintain that medical competition within the boundaries of legally qualified practice is producing for the public the best results of which it is capable, and that its legitimate limits are thus reached. Let us add a few sentences in proof of this contention. A prolonged study of, and a wide familiarity with, all the circumstances of medical life justifies the assertion that medical education is of so free and various a character as to admit men of all ranks to its curricula and its legal qualifications. Hardly any man is so poor in purse or general culture but that he may force his way with a little energy and perseverance into one or other of our numerous medical schools; and through that school he may pass by means of one of the many examining boards into the sphere of legitimate and recognised practice. The proof of this is obvious on all hands. Men of every variety of natural capacity, of every order of literary culture, and of every degree of social training and fitness are to be actually found in the ranks of the medical profession. Not only so, but they are to be found in numbers not only so large as to secure the most strenuous competition, but to bring about the absolute suppression and extinction of all but the very fittest. Of medical students there are hundreds every year who begin the medical curriculum but never proceed to its close, and even of those who pass all the examinations and persevere until a legal qualification is obtained there are large numbers annually who drop from our ranks, and seek elsewhere relief from that dreaded starvation with which the keen competition within medicine has so ruthlessly threatened them.

For these two reasons then, that in actual scientific work the quack cannot in any sense ever be a real competitor with the legally qualified medical man, and that within the ranks of legally qualified practice all the advantages that the sternest competition can confer upon the public are already secured, we welcome most heartily the decision of the General Medical Council to take an active part in the suppression of fraudulent quackery, and we ask the public to give to the Council its approval and its practical help.

